# Indocyanine Green Sentinel Lymph Node Mapping as a Tool for Personalized Surgical Management in Uterine Corpus Cancer: A Single-Center Comparative Study

**DOI:** 10.3390/jpm16030168

**Published:** 2026-03-18

**Authors:** Krzysztof Nowak, Wiktor Bek, Maja Mrugała, Zofia Borowiec, Ewa Milnerowicz-Nabzdyk

**Affiliations:** Oncological Gynecology Clinical Department, Centre of Oncology, 45-061 Opole, Poland

**Keywords:** indocyanine green, sentinel lymph node mapping, endometrial cancer, staging, obesity, real-world data

## Abstract

**Objectives**: This study aimed to investigate the usefulness and safety of sentinel lymph node (SLN) mapping in comparison to other types of lymph node dissection in patients with uterine corpus cancers. **Methods**: Retrospective data from 161 patients subjected to uterine corpus cancer staging with SLN mapping with indocyanine green (ICG) dye were collected. **Results**: SLN procedure was associated with a complication rate of 0%, a median number of dissected lymph nodes of 2 (range 0–13), and a median hospitalization following surgery of 5 (range:2–23) days. Systemic lymphadenectomy and one-sided pelvic lymph node resection were associated with the highest percentage of complications (12% and 25%; *p* = 0.0030), while the post-surgery course was uneventful for the selective lymphadenectomy group and SLN. Complication rates were the highest in patients with obesity and severe obesity (5.1% and 9.1%, respectively). The number of lymph nodes resected dropped numerically with increasing BMI. Successful ICG injection and SLN mapping were significantly more frequent in SLN procedures. **Conclusions**: Our study showed that SLN mapping was characterized by a low complication rate and short hospitalization following surgery, and obesity appeared to be related to a higher complication rate. Tailored surgical strategies and individualized patient selection are crucial for the success of SLN mapping; therefore, factors associated with successful SLN mapping with ICG need further exploration.

## 1. Introduction

Endometrial cancer originates from cells in the uterus, most commonly from the endometrium. Uterine corpus cancer is the second most prevalent cancer in women globally, following breast cancer [1]. The American Cancer Society reports that the annual prevalence of uterine corpus cancers has increased by about 1% among women aged 50 and older since the mid-2000s and by almost 2% among younger women since at least the mid-1990s. In addition to the rising incidence of uterine corpus cancer, mortality rates from this cancer are also increasing at a rate of approximately 1% per year [2]. Abnormal uterine bleeding allows for early diagnosis in most cases of endometrial cancer. However, other symptoms have not been sufficiently examined to determine their diagnostic accuracy, potentially leading to delayed diagnosis of uterine corpus cancers [3].

Staging is a crucial diagnostic process used to determine the extent of cancer spread and to guide the subsequent treatment pathway. The FIGO staging of endometrial cancer is based on several malignant process features that include histological types, tumor patterns, and molecular classification. The evaluation of the invasion of lymphovascular space and lymph node status is also included in the assessment of cancer spread [4]. In FIGO IIIC involvement of pelvic and para-aortic lymph nodes occurs, including micrometastasis (lesions of 0.2–2 mm in size and/or those of more than 200 cells) and macrometastasis (lesions larger than 2 mm). To determine the risk of nodal metastasis and the necessity of lymphadenectomy, an intraoperative frozen section of dissected lymph nodes is performed. However, this procedure lacks sufficient diagnostic accuracy. Sentinel lymph node (SLN) mapping using indocyanine green (ICG) with near-infrared imaging and ultra-staging serves as a less invasive alternative that can provide additional information on nodal status [5]. SLN mapping has become more widely used in clinical practice as it offers benefits to patients. Numerous recent meta-analyses have demonstrated the utility of SLN assessment in cancer staging [6,7,8]. However, some critics have questioned the reliability of these meta-analyses, particularly those evaluating the role of SLN in staging endometrial cancer [9]. Despite these concerns, SLN assessment offers significant advantages over lymphadenectomy and is steadily gaining popularity. This underscores the need for gathering more evidence on the usefulness of SLN mapping in uterine corpus cancers.

Researchers highlight several advantages of SLN mapping, including accurate detection of nodal metastases and decreased operative and postoperative morbidity. It also presents a lower risk of intraoperative adverse events and long-term complications such as lymphedema, which can significantly impact quality of life [10]. However, some pitfalls can lead to failure of the procedure. Reasons for SLN mapping failure include lymphatic obstruction by tumor and clinically enlarged lymph nodes, advanced cancer stage, excessive body weight of the patient, menopausal status, and prior gynecological or pelvic surgeries [11,12]. Although the evidence for SLN mapping with ICG in endometrial cancer is encouraging and fluorescent imaging has emerged, the Society of Gynecologic Oncology’s call for more high-quality evidence remains valid. There are still gaps in the existing evidence and a lack of SLN mapping algorithms validated specifically for uterine corpus cancer. Therefore, we conducted this single-center retrospective study to present experience from our institution. The aim of the study was to investigate the usefulness and safety of SLN mapping in comparison to other types of lymph node dissection in patients with uterine corpus cancers.

## 2. Materials and Methods

### 2.1. Study Design

This retrospective single-center study was conducted at the Oncological Gynecology Clinical Department, Centre of Oncology in Opole, Poland. All data were retrospectively collected from our institution’s medical records. Data were retrieved for the period from 1 January 2022 to 31 December 2023. All authors were involved in both the conduct of surgical procedures and the collection of data. The study was approved by the Bioethics Committee.

### 2.2. Patients and Patient Data

In total, 161 patients subjected to lymph node dissection in the course of uterine corpus cancer between 2022 and 2023 were included. This study included all women treated in our institution during the analyzed period who had data on age, body mass index (BMI), and lymph node dissection in the course of uterine corpus cancer. Data related to surgery included the type of lymphadenectomy, number of dissected lymph nodes, type of hysterectomy, the use of ICG, duration of hospitalization, information about complications, and pre- and postoperative hemoglobin levels. Complications were captured during postoperative follow-up visits at 2 and 4 weeks after surgery, covering intraoperative and early postoperative events. No other eligibility criteria were applied. For the purpose of uterine cancer staging, the FIGO methodology was used [4].

As obesity is considered a risk factor for SLN mapping, patients were categorized into four BMI categories based on the WHO recommendations. A BMI between 25 and 29.9 kg/m^2^ was used for overweight, A BMI between 30 and 39.9 kg/m^2^ was used for obesity, whereas a BMI of 40 kg/m^2^ and higher was used for severe obesity [13].

### 2.3. SNL Mapping and ICG Procedure

For SLN mapping, we used a laparoscopic procedure, in which ICG was used. ICG is a near-infrared (NIR) fluorescent dye. Its absorption spectrum depends on the solvent and dye concentration; however, its peak is about 800 nm. ICG solutions in plasma and distilled water are stable for at least 4 h [14]. It was first approved by the US Food and Drug Administration (FDA) in 1959 to enhance the visualization of vasculature and tissue structures during surgical procedures [15,16]. Currently, it is approved by the FDA for visual assessment of vessels and major extra-hepatic bile ducts during minimally invasive surgeries [16]. Lymph node mapping using ICG is considered an off-label use of the dye [17].

**Figure 1:**
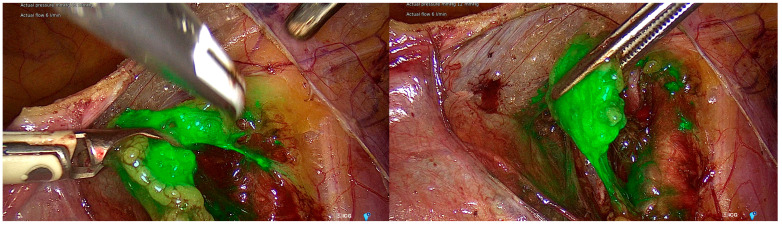
Representative intraoperative images of indocyanine green (ICG)-guided lymph node mapping.

We used a commercial preparation of ICG (Merck Life Science, Poznań, Poland). A vial contains 25 mg of ICG (2.5 mg of dye per mL) and requires reconstitution. The manufacturer recommends an ICG dose of 0.02 mL/kg to be scaled to the patient’s weight, followed by a 10 mL saline bolus. This provides 0.05 mg/kg of ICG. Multiple doses can be administered as needed, up to a maximum of 2 mg/kg of patient body weight. Vessels become visible 5–30 s after administration, with visibility lasting up to 20–30 s. For organs, visibility starts at 20–30 s and can last up to 20–120 min. ICG has a half-life of 150 to 180 s and is exclusively removed from circulation by the liver to bile [16]. In our institution, ICG is used at a standard concentration of 1.25 mg/mL, with a total injected volume of 4 mL. The tracer is administered into the cervix at the 3 and 9 o’clock positions, with 1 mL injected deeply into the cervical stroma and 1 mL injected superficially (submucosal) at each site. Injection is performed immediately before abdominal access and camera insertion. Visualization is achieved using a RUBINA^®^ system with a 0-degree laparoscope (KARL STORZ SE & Co. KG, Tuttlingen, Germany). Figure 1 shows intraoperative visualization of lymph nodes using ICG.

All patients were operated on by authors who are experienced gynecologic surgeons. In our institution, ICG is injected via cervical injection as recommended by the Society of Gynecologic Oncology Consensus Guidelines, as this technique is simple and achieves the highest SLN detection rates [17]. During lymph node mapping, the ICG solution is slowly injected into the superficial stroma of the cervix to enhance the accumulation of ICG in lymph nodes and reduce staining of deep pelvic tissues. After an ICG injection, a complete evaluation of the peritoneal cavity is conducted. SLN dissection starts by assessing the retroperitoneal spaces and identifying the sentinel drainage pathways from the parametria. This is followed by the excision of the most proximal lymph nodes in these sentinel pathways. Suspicious lymph nodes are dissected regardless of SLN mapping results. In case of mapping failure, a hemi-pelvic side-specific lymphadenectomy was attempted [18].

At our institution, the SLN mapping procedure is performed to identify and dissect the lymph nodes at the greatest risk for metastasis. This approach limits the extent of systemic lymphadenectomy and reduces associated morbidity. Lymphatic obstruction by cancer lesions and obesity are considered risk factors that hinder the success of the SLN procedure [17]. If the SLN mapping procedure fails, lymph nodes are mapped only in specific regions. Additionally, if the operator identifies suspicious, enlarged, or firm lymph nodes on visual or palpation examination, an extended lymph node resection is performed, including systemic lymphadenectomy, regardless of the SLN mapping results.

### 2.4. Statistical Analysis

Data were presented as means with standard deviation, medians, ranges, numbers, and percentages depending on the type of data distribution. The normality of continuous variables was checked with the Shapiro–Wilk test. Differences between the two groups were compared with the Student *t*-test (paired for repeated measures). When three groups or more groups were compared the one-way ANOVA with the Scheffé test for pairwise comparisons or its nonparametric equivalent test (Kruskal–Wallis test with the Conover test for post hoc comparison) was employed. Categorical variables were compared using contingency tables and the chi-squared test. The differences were considered statistically significant at a *p*-value < 0.05 for all tests performed. The collected data were stored in an Excel spreadsheet (Microsoft 365, Version 2510; Microsoft Corp., Redmond, WA, USA) and statistically analyzed using MedCalc v. 19.5.3 (MedCalc Software Ltd., Ostend, Belgium).

## 3. Results

Between 2022 and 2023, 161 patients were qualified for uterine corpus cancer resection in our institution. On average, patients were 65.65 ± 9.91 years old with a BMI ranging from 18 to 52 (mean 32.58 ± 7.46). Of 154 patients with BMI data, 24 (15.6%) had a healthy weight, 34 (22.1%) were overweight, 70 (45.5%) were obese, and 25 (16.2%) were severely obese. Of the study group, 133 patients underwent lymphadenectomy or SLN resection. This procedure was abandoned in 28 patients due to the following reasons: disseminated malignant process, unresectable lesion, cytoreduction, and the extent of the surgery. The patient characteristics are presented in Table 1.

Complications following lymph node resection were analyzed in patients who underwent any type of lymphadenectomy or SLN resection. Complications developed in six (4.5%) patients and included lymphorrhea, lymphocele, and vesicovaginal fistula. The post-surgery course was uneventful in the selective lymphadenectomy group and SLN resection group, while systemic lymphadenectomy and one-sided pelvic lymph node resection were associated with the highest percentage of complications. The difference in proportions was statistically significant.

The hemoglobin measurements were characterized by a normal distribution. The mean hemoglobin level before surgery was 13.31 ± 1.36, and after surgery, it was 11.60 ± 1.30 (*p* < 0.0001). When comparing the pre- and post-surgery differences in hemoglobin levels between lymphadenectomy type groups, no significant differences were detected (*p* = 0.0520), although the smallest numerical drop was observed for the SLN procedure.

Hospitalization was counted as the number of hospital stay days after surgery; however, the length of hospital stay depended on many factors, not only the procedure, including the overall health condition of the patient, in-hospital procedures, and urgency to perform surgery. The shortest procedures were conducted as a one-day surgery. This variable was characterized by a non-normal distribution (mean = 5.83 ± 3.94; median = 5). Differences between groups were statistically significant (*p* = 0.0003). Selective lymphadenectomy was associated with significantly longer hospitalization (median 9 days) in comparison to SLN, pelvic one-sided SLN, and pelvic one-sided lymphadenectomy. The shortest hospitalization was reported for patients undergoing one-sided pelvic SLN resection, with a median duration of 4.5 days. This was significantly shorter compared to those undergoing selective or systemic lymphadenectomy.

The number of lymph nodes resected was characterized by a non-normal distribution (mean 8.54 ± 10.44; median = 3). The differences between groups were statistically significant (*p* < 0.0001). The highest numbers were observed during systemic and one-sided pelvic lymphadenectomy; both were significantly higher compared to selective lymphadenectomy, one-sided pelvic SLN, and SLN.

BMI was characterized by a normal distribution. The only significant difference between groups was observed for SLN and systemic lymphadenectomy. Other comparisons showed that the differences between groups were not significant. Table 2 presents the summary characteristics for complications, pre- and postoperative differences in the hemoglobin levels, duration of hospitalization, the number of lymph nodes resected, and BMI across patients stratified by type of surgery.

The additional analysis compared the number of lymph nodes resected and the complication rates among different BMI categories. Although the percentage of complicated cases was the highest in severely obese patients, differences between groups were not statistically significant. It is worth noting that almost all complications occurred in patients with obesity. Regarding the number of resected lymph nodes, it can be noted that the number of lymph nodes resected dropped numerically with increasing BMI. Significant differences were detected when comparing groups with a healthy weight and overweight with groups with obesity and severe obesity. Results for patients stratified by BMI category are shown in Table 3.

ICG was used in 104 patients. Successful ICG was the most common in the SLN group and pelvic one-sided SLN, while the least successful ICG was in the pelvic one-sided lymphadenectomy, selective lymphadenectomy, and systemic lymphadenectomy. The differences between the groups were significant. The use of ICG in the study group is reported in Table 4.

After surgery, staging was changed in 38 cases with a similar rate in each group (*p* = 0.9286). The proportions of changes in the staging after surgery are depicted in Table 5. Initial staging was I in 122 patients, II in 2 patients, III in 3 patients, and IV in 4 patients, which was confirmed or modified during surgery into I in 105 patients, II in 8 patients, III in 16 patients, and 4 in 3 patients.

## 4. Discussion

This study aimed to investigate the usefulness of SLN mapping in a group of patients with uterine corpus cancer. We found that SLN procedures were associated with lower complication rates, a smaller number of dissected lymph nodes, and shorter hospitalization following surgery, but a similar drop in hemoglobin levels in comparison to more invasive procedures. Obesity was associated with a higher rate of complications, which were the highest in patients with obesity and severe obesity (5.1% and 9.1%, respectively) and lower numbers of dissected lymph nodes. Successful ICG injection and lymph node mapping were significantly more frequent in SLN procedures.

Lymphadenectomy aids in disease staging, while the evaluation of SLN helps determine the extent of cancer spread and guides the subsequent treatment strategy. Recently, several meta-analyses were published on the usefulness of SLN evaluation in endometrial cancer. A meta-analysis conducted by Yao et al. [6] aimed to investigate how SLN assessment and lymphadenectomy impact the prognosis of patients with advanced endometrial cancer. The analysis included 492 patients undergoing SLN and 6689 patients undergoing lymphadenectomy. The difference in overall survival between the groups was not significant, leading to the conclusion that SLN assessment can be used as an alternative to lymphadenectomy in the treatment of patients with advanced endometrial cancer. Wang and Liu [7] devoted their meta-analysis to the usefulness of laparoscopy SLN mapping in endometrial cancer. Their study included 389 patients undergoing bilateral SLN detection participating in 8 studies. The overall detection rate based on SLN mapping was 96.3%, with a sensitivity of 73.1%. The biggest meta-analysis was conducted by Kang et al. [8] and included 26 studies and 1101 SLN mapping procedures. This study aimed to investigate the diagnostic performance of SLN mapping in endometrial cancer. The meta-analysis reveals a detection rate of 78% and a sensitivity of 93%. The authors concluded that although SLN mapping demonstrates good diagnostic performance, most data on the procedure come from small studies with heterogeneous populations. Therefore, the results should be interpreted with caution. Some other meta-analyses echo the results of the usefulness of the SLN mapping in endometrial cancer [19,20]; however, there are also critical voices [9].

One of the first prospective studies on the use of ICG in patients with low-grade endometrial cancer was the FIRES trial. In this study, Rossi et al. [21] enrolled 385 patients with endometrial cancer in whom a standardized injection of ICG and lymph node mapping was followed by pelvic lymphadenectomy with or without para-aortic lymphadenectomy. Most patients in this study had low-grade endometrial cancer (postoperative stage IA, n = 228; 66%). The study found that SLN mapping using ICG demonstrated a sensitivity of 97.2% (95% CI: 85.0–100) for detecting node-positive disease, with a negative predictive value of 99.6% (95% CI: 97.9–100). The authors concluded that ICG is characterized by high diagnostic accuracy in detecting metastases from endometrial cancer, and thus it can effectively substitute lymphadenectomy for staging purposes. While sentinel lymph node biopsy may miss metastases in 3% of patients with node-positive disease, it also has the potential to reduce the morbidity associated with complete lymphadenectomy in a significant number of patients. Conversely, Pelvic Sentinel lymph node detection in high-risk endometrial cancer (SHREC-trial) [22] included women with more advanced endometrial cancer. Persson et al. [22] recruited 257 women with at least one preoperative high-risk criterion, including endometrioid cancer FIGO grade III, non-endometrioid histology, over 50% myometrial tumor invasion, cervical stromal invasion, or a non-diploid cytometry. The study found that SLN mapping using ICG demonstrated a sensitivity of 98% (95% CI: 89–100) and a negative predictive value of 99.5% (95% CI: 97–100). The sensitivity of the overall SLN algorithm was 100% (95% CI: 92–100), and the negative predictive value was 100% (95% CI: 98–100). The authors concluded that using an SLN algorithm under the guidance of an experienced surgeon can effectively rule out nodal involvement in 99% of cases, making it a safe alternative to a complete lymphadenectomy in high-risk endometrial cancer. Those promising and encouraging results have inspired other researchers to contribute their experiences with SLN mapping. As a result, SLN mapping has now become the standard approach in surgical staging for endometrial cancer [10].

Complications reported in the literature during and after SLN mapping are rare. Our study showed that SLN mapping was uneventful with no adverse events related to this procedure reported. On the other hand, systemic lymphadenectomy was associated with three (12%) complications; namely, there were two cases of lymphorrhea and one lymphocele. The limitation of the systemic lymphadenectomy group can be the number of less representative cases than for the SLN group, but much less indication nowadays exists to perform such a procedure in endometrial cancer due to new ESGO recommendations. For this reason, the number of complications can be less objective. Furthermore, there is a scarcity of data on complication rates in patients with systemic lymphadenectomy. The FIRES trial [21] documented just one adverse event associated with SLN mapping out of 385 patients, specifically a ureteral injury during SLN dissection. Conversely, no adverse events were reported among the 257 participants in the SHREC trial [22]. In the systematic review and meta-analysis conducted by Bodurtha et al. [20], 7 of 55 included studies reported serious complications after completing lymphadenectomy following SLN mapping (five lymphoceles, two vascular injuries). Their literature search did not identify any study that compared the risk of lymphedema among patients undergoing SLN mapping alone in comparison to systemic lymphadenectomy. Additionally, while several meta-analyses on SLN mapping are available in the literature, they predominantly emphasize efficacy rather than the safety of the procedure [7,23,24].

Obesity is a health issue that increases the risk of developing uterine cancers and is also associated with a higher likelihood of medical procedure failures [2]. Furthermore, there is a high prevalence of obesity among women with uterine cancer, as confirmed also in our study [25]. The WHO highlights the alarming global trend of rapid weight gain and recommends using BMI to categorize individuals into different weight categories, which are associated with an increased risk of various diseases [26]. The Global Burden of Disease Study showed that high BMI caused 4.7 million deaths globally contributing to increased health care expenses [27,28]. The Society of Gynecologic Oncology lists obesity among suggested reasons for SNL mapping failure [17]. Eriksson et al. [29] specifically investigated the impact of obesity on the success rate of SLN mapping procedures in patients with uterine cancer undergoing robotic surgery. Researchers retrospectively analyzed the use of blue dye or ICG with near-infrared fluorescence imaging in 472 study participants. The study demonstrated a significant decrease in the rate of successful bilateral mapping for both the ICG (*p* < 0.001) and blue dye groups (*p* = 0.041) with increasing BMI. Additionally, obesity was significantly associated with longer procedure times. Tanner et al. [12] investigated factors associated with successful bilateral SLN mapping in endometrial cancer. The study included 111 women, of whom 80 (85%) suffered from low-grade cancer. A BMI greater than 30 was considered a potential risk factor for failed mapping. However, the use of ICG was particularly beneficial in this group of patients [12]. Our experience showed that although the percentage of complications was highest in severely obese patients, the differences between groups were not statistically significant. This may be due to the small size of the study group and the low frequency of complications.

In the future, the wide use of ICG in endometrial cancer surgery could significantly enhance the precision of lymph node mapping, thereby improving the accuracy of staging and reducing the need for extensive lymphadenectomies. However, there are still gaps in the existing evidence and a lack of SLN mapping algorithms validated specifically for endometrial cancer. The Consensus Recommendations from the Society of Gynecologic Oncology acknowledge the need for future studies to compare patterns of disease recurrence in patients with high-risk histologies. These studies should evaluate the efficacy of using the SLN algorithm alone, without completion of lymphadenectomy, against traditional lymphadenectomy [17]. Additionally, ongoing advancements in ICG imaging technology may improve real-time visualization, potentially leading to more effective and less invasive surgical interventions. Advancements in SLN mapping technology are exemplified by the FILM study, a randomized, prospective, open-label, multicenter study specifically devoted to the PINPOINT near-infrared fluorescence imaging system (Stryker, Kalamazoo, MI, USA) [25]. The trial aimed to compare ICG with isosulfan blue dye in detecting sentinel lymph nodes in women with cervical and uterine cancers. The study demonstrated that ICG was not inferior. However, the study population was heterogeneous, including patients with both endometrial and cervical cancer. Data from the literature are often used to analyze gynecologic cancers collectively. The recent meta-analysis by Chauvet et al. [30] published this year on the use of indocyanine green in gynecologic surgery, did not specifically focus on endometrial cancer. Chauvet et al. [30] included 147 studies involving gynecologic surgery, of which only 6 were randomized controlled trials. Among these, 52 focused on endometrial cancer, involving a total of 6216 patients. These numbers highlight that, despite the growing evidence on ICG use in endometrial cancer, further research is still needed. The researchers acknowledge that the analyzed studies involved diverse patient populations. ICG was injected during different types of surgeries, at various sites, and with varying volumes and concentrations of ICG doses. Additionally, the outcomes were defined differently. These variations hinder comparison between studies and make it difficult to determine which algorithm is the most effective and safest. Additionally, the meta-analysis focused on detection rates overall, by site of injection, and ICG concentration. The overall ICG detection rate for endometrial cancer was 95.2% (95% CI [93.2–96.8%]). To our knowledge, no meta-analysis has been conducted to evaluate the safety or application algorithm. Although the evidence is very encouraging and fluorescent imaging has emerged as the most consistently effective technique for pelvic SLN detection in endometrial cancer [23,31,32,33], the Society of Gynecologic Oncology’s call for more high-quality evidence remains valid [17].

When interpreting the results, it is important to take into account several limitations of this study. This retrospective study included participants from existing hospital medical records, making the study population non-representative. Also, historical data are characterized by lower availability and accuracy in comparison to data collected prospectively, which can potentially impact the study findings. The study population is heterogeneous. Although heterogeneity poses challenges in terms of interpretation and generalizability, it also presents real-world data that match the settings of clinical practice. Finally, we did not analyze all determinants influencing complication rates or procedure selection, nor did we model surgeon-level effects. Our analysis was intended to present the experience of a single center and to describe the procedural outcomes observed in our clinical practice. Predefined allocation or planning to ensure balanced subgroup representation should be taken into account when designing future prospective studies.

## 5. Conclusions

Due to the rising incidence and increasing mortality rates of uterine corpus cancer, there is a critical need for early detection, accurate staging, and effective treatment. In our center, SLN mapping performed with ICG was characterized by a low complication rate and short hospitalization following surgery, while a comparable drop in hemoglobin level was observed in more invasive procedures. Obesity appeared to be related to a higher complication rate.

Tailored surgical strategies and individualized patient selection are crucial for the success of SLN mapping; therefore, factors associated with successful SLN mapping with ICG need further exploration.

## Figures and Tables

**Table 1:** Patient characteristics by lymph node resection type, covering age and BMI.

	Abandoned	Pelvic, One-Sided	Selective	SLN	SLN, Pelvic, One-Sided	Systemic
Patient number	28	8	5	85	10	25
Age, mean ± SD	68.04 ± 10.50	69.88 ± 6.33	69.20 ± 5.17	64.93 ± 10.56	63.50 ± 8.09	64.20 ± 8.84
Age, range	44–93	61–80	62–76	31–83	49–73	46–80
BMI, mean ± SD	33.57 ± 8.10	31.68 ± 4.45	27.49 ± 6.99	33.63 ± 7.31	36.09 ± 7.18	28.09 ± 6.49
BMI, range	22–50	22–36	20–37	19–52	26–52	18–46
Healthy weight	5 (21.7%)	1 (12.5%)	2 (40.0%)	8 (9.6%)	(0.00%)	8 (32.0%)
Overweight	3 (13.0%)	0 (0.00%)	1 (20.00%)	18 (21.7%)	2 (20.0%)	10 (40.0%)
Obese	11 (47.8%)	7 (87.5%)	2 (40.0%)	39 (47.0%)	6 (60.00%)	5 (20.0%)
Severely obese	4 (17.4%)	(0.00%)	(0.00%)	18 (21.7%)	2 (20.0%)	2 (8.00%)

BMI, body mass index; SD, standard deviation; SLN, sentinel lymph node.

**Table 2:** Patient characteristics by lymph node resection type covering complications, hemoglobin level, number of resected lymph nodes, and mean BMI.

	Pelvic, One-Sided ^1^	Selective ^2^	SLN ^3^	SLN, Pelvic, One-Sided ^4^	Systemic ^5^	*p* Value
Complications	2 (25%)	0 (0%)	0 (0%)	1 (10%)	3 (12%)	0.0030 *
Difference in Hb, mean ± SD	2.10 ± 0.78	2.23 ± 0.31	1.52 ± 0.87	1.97 ± 1.10	2.01 ± 1.01	0.0520 ^†^
Length of stay, median days (range)	4.5 (3–13) ^2^	9 (6–22) ^1,3,4^	5 (2–23) ^2,5^	4.5 (2–8) ^2,5^	6 (2–31) ^3,4^	0.0003 ^§^
Lymph nodes resected, median (range)	21 (3–36) ^2,3,4^	6 (1–20) ^1,5^	2 (0–13) ^1,4,5^	6 (2–27) ^1,3,5^	21 (5–48) ^2,3,4^	<0.0001 ^§^
BMI, mean ± SD	31.68 ± 4.45	27.49 ± 6.99	33.63 ± 7.31 ^5^	36.09 ± 7.18	28.09 ± 6.49 ^3^	0.0030 ^†^

Hb, hemoglobin. * chi-squared test; ^†^ one-way ANOVA; ^§^ the Kruskal–Wallis test. ^1,2,3,4,5^ Column numbering allows showing significant differences (*p* < 0.05) for the respective comparisons in the post hoc analysis.

**Table 3:** Complication rates and numbers of dissected lymph nodes by BMI category.

	Healthy Weight ^1^	Overweight ^2^	Obese ^3^	Severely Obese ^4^	*p* Value
Complications	1 (5.3%)	0 (0.00%)	3 (5.1%)	2 (9.1%)	0.4633 *
Lymph nodes resected, median (range)	8 (1–38) ^3,4^	5 (2–48) ^3,4^	3 (0–36) ^1,2^	2 (0–27) ^1,2^	0.0042 ^†^

* chi-squared test; ^†^ Kruskal–Wallis test. ^1,2,3,4^ Column numbering allows showing significant differences (*p* < 0.05) for the respective comparisons in the post hoc analysis.

**Table 4:** The use of ICG for SLN mapping in the study group.

	Pelvic, One-Sided	Selective	SLN	SLN, Pelvic, One-Sided	Systemic	*p* Value
ICG						<0.0001 *
Yes	2 (25%)	1 (20%)	84 (98.8%)	10 (100%)	7 (28.0%)	
Failed	6 (75%)	4 (80.0%)	1 (1.2%)	0 (0.00%)	18 (72.0%)	

ICG, indocyanine green; * chi-squared test.

**Table 5:** Changes in staging after surgery in the study group.

	Pelvic, One-Sided	Selective	SLN	SLN, Pelvic, One-Sided	Systemic	*p* Value
Staging						0.9286 *
Changed	2 (25%)	1 (20%)	24 (28.2%)	4 (40.0%)	7 (29.2%)	
The same	6 (75%)	4 (80%)	61 (71.8%)	6 (60.0%)	17 (70.8%)	

* chi-squared test.

## Data Availability

The data that support the findings of this study are available upon request from the corresponding author. Figshare with: https://doi.org/10.6084/m9.figshare.29200268.

## References

[1] Miller K.D., Nogueira L., Devasia T., Mariotto A.B., Yabroff K.R., Jemal A., Kramer J., Siegel R.L. (2022). Cancer treatment and survivorship statistics, 2022. CA Cancer J. Clin..

[2] Siegel R.L., Miller K.D., Wagle N.S., Jemal A. (2023). Cancer statistics, 2023. CA Cancer J. Clin..

[3] Boeckstaens S., Dewalheyns S., Heremans R., Vikram R., Timmerman D., Bosch T.V.D., Verbakel J.Y. (2020). Signs and symptoms associated with uterine cancer in pre- and postmenopausal women. Heliyon.

[4] Berek J.S., Matias-Guiu X., Creutzberg C., Fotopoulou C., Gaffney D., Kehoe S., Lindemann K., Mutch D., Concin N. (2023). FIGO staging of endometrial cancer: 2023. Int. J. Gynaecol. Obstet..

[5] Blakely M., Liu Y., Rahaman J., Prasad-Hayes M., Tismenetsky M., Wang X., Nair N., Dresser K.A., Nagarsheth N., Kalir T. (2019). Sentinel Lymph Node Ultra-staging as a Supplement for Endometrial Cancer Intraoperative Frozen Section Deficiencies. Int. J. Gynecol. Pathol..

[6] Yao H.M., Luo R.M., Tong R.M., Wei Y.M., Zheng K.M., Hu X. (2023). Impact of sentinel lymph node assessment on the outcomes of patients with advanced endometrial cancer: A meta-analysis. Medicine.

[7] Wang L., Liu F. (2018). Meta-analysis of laparoscopy sentinel lymph node mapping in endometrial cancer. Arch. Gynecol. Obstet..

[8] Kang S., Yoo H.J., Hwang J.H., Lim M.-C., Seo S.-S., Park S.-Y. (2011). Sentinel lymph node biopsy in endometrial cancer: Meta-analysis of 26 studies. Gynecol. Oncol..

[9] Mariño M.A.G. (2022). Sentinel Lymph Node Biopsy in Endometrial Cancer—A Systematic Review and Quality Assessment of Meta-Analyses. Rev. Bras. Ginecol. Obstet..

[10] Salman L., Cusimano M.C., Marchocki Z., Ferguson S.E. (2024). Sentinel lymph node mapping in endometrial cancer: Current evidence and practice. J. Surg. Oncol..

[11] Barlin J.N., Khoury-Collado F., Kim C.H., Leitao M.M., Chi D.S., Sonoda Y., Alektiar K., DeLair D.F., Barakat R.R., Abu-Rustum N.R. (2012). The importance of applying a sentinel lymph node mapping algorithm in endometrial cancer staging: Beyond removal of blue nodes. Gynecol. Oncol..

[12] Tanner E.J., Sinno A.K., Stone R.L., Levinson K.L., Long K.C., Fader A.N. (2015). Factors associated with successful bilateral sentinel lymph node mapping in endometrial cancer. Gynecol. Oncol..

[13] National Health Servise What Is the Body Mass Index (BMI)?. https://www.nhs.uk/common-health-questions/lifestyle/what-is-the-body-mass-index-bmi/.

[14] Landsman M.L., Kwant G., Mook G.A., Zijlstra W.G. (1976). Light-absorbing properties, stability, and spectral stabilization of indocyanine green. J. Appl. Physiol..

[15] Starosolski Z., Bhavane R., Ghaghada K.B., Vasudevan S.A., Kaay A., Annapragada A. (2017). Indocyanine green fluorescence in second near-infrared (NIR-II) window. PLoS ONE.

[16] Drugs.com ICG for Injection Set Prescribing Information. https://www.drugs.com/pro/icg-for-injection-set.html.

[17] Holloway R.W., Abu-Rustum N.R., Backes F.J., Boggess J.F., Gotlieb W.H., Lowery W.J., Rossi E.C., Tanner E.J., Wolsky R.J. (2017). Sentinel lymph node mapping and staging in endometrial cancer: A Society of Gynecologic Oncology literature review with consensus recommendations. Gynecol. Oncol..

[18] Khoury-Collado F., Glaser G.E., Zivanovic O., Sonoda Y., Levine D.A., Chi D.S., Gemignani M.L., Barakat R.R., Abu-Rustum N.R. (2009). Improving sentinel lymph node detection rates in endometrial cancer: How many cases are needed?. Gynecol. Oncol..

[19] Lin H., Ding Z., Kota V.G., Zhang X., Zhou J. (2017). Sentinel lymph node mapping in endometrial cancer: A systematic review and meta-analysis. Oncotarget.

[20] Smith A.J.B., Fader A.N., Tanner E.J. (2017). Sentinel lymph node assessment in endometrial cancer: A systematic review and meta-analysis. Am. J. Obstet. Gynecol..

[21] Rossi E.C., Kowalski L.D., Scalici J., Cantrell L., Schuler K., Hanna R.K., Method M., Ade M., Ivanova A., Boggess J.F. (2017). A comparison of sentinel lymph node biopsy to lymphadenectomy for endometrial cancer staging (FIRES trial): A multicentre, prospective, cohort study. Lancet Oncol..

[22] Persson J., Salehi S., Bollino M., Lönnerfors C., Falconer H., Geppert B. (2019). Pelvic Sentinel lymph node detection in High-Risk Endometrial Cancer (SHREC-trial)-the final step towards a paradigm shift in surgical staging. Eur. J. Cancer.

[23] How J.A., O’Farrell P., Amajoud Z., Lau S., Salvador S., How E., Gotlieb W.H. (2018). Sentinel lymph node mapping in endometrial cancer: A systematic review and meta-analysis. Minerva Ginecol..

[24] Raffone A., Fanfani F., Raimondo D., Rovero G., Renzulli F., Travaglino A., De Laurentiis U., Santoro A., Zannoni G.F., Casadio P. (2023). Predictive factors of sentinel lymph node failed mapping in endometrial carcinoma patients: A systematic review and meta-analysis. Int. J. Gynecol. Cancer.

[25] Frumovitz M., Plante M., Lee P.S., Sandadi S., Lilja J.F., Escobar P.F., Gien L.T., Urbauer D.L., Abu-Rustum N.R. (2018). Near-infrared fluorescence for detection of sentinel lymph nodes in women with cervical and uterine cancers (FILM): A randomised, phase 3, multicentre, non-inferiority trial. Lancet Oncol..

[26] Cibula D., Raspollini M.R., Planchamp F., Centeno C., Chargari C., Felix A., Fischerová D., Jahnn-Kuch D., Joly F., Kohler C. (2023). ESGO/ESTRO/ESP Guidelines for the management of patients with cervical cancer—Update 2023. Int. J. Gynecol. Cancer.

[27] Dai H., Alsalhe T.A., Chalghaf N., Riccò M., Bragazzi N.L., Wu J. (2020). The global burden of disease attributable to high body mass index in 195 countries and territories, 1990–2017: An analysis of the Global Burden of Disease Study. PLoS Med..

[28] Hecker J., Freijer K., Hiligsmann M., Evers S.M.A.A. (2022). Burden of disease study of overweight and obesity; the societal impact in terms of cost-of-illness and health-related quality of life. BMC Public Health.

[29] Eriksson A.G.Z., Montovano M., Beavis A., Soslow R.A., Zhou Q., Abu-Rustum N.R., Gardner G.J., Zivanovic O., Barakat R.R., Brown C.L. (2016). Impact of Obesity on Sentinel Lymph Node Mapping in Patients with Newly Diagnosed Uterine Cancer Undergoing Robotic Surgery. Ann. Surg. Oncol..

[30] Chauvet P., Jacobs A., Jaillet L., Comptour A., Pereira B., Canis M., Bourdel N. (2024). Indocyanine green in gynecologic surgery: Where do we stand? A literature review and meta-analysis. J. Gynecol. Obstet. Hum. Reprod..

[31] Morales-Conde S., Licardie E., Alarcón I., Balla A. (2022). Indocyanine green (ICG) fluorescence guide for the use and indications in general surgery: Recommendations based on the descriptive review of the literature and the analysis of experience. Cir. Esp. (Engl. Ed.).

[32] Jewell E.L., Huang J.J., Abu-Rustum N.R., Gardner G.J., Brown C.L., Sonoda Y., Barakat R.R., Levine D.A., Leitao M.M. (2014). Detection of sentinel lymph nodes in minimally invasive surgery using indocyanine green and near-infrared fluorescence imaging for uterine and cervical malignancies. Gynecol. Oncol..

[33] Bogani G., Ditto A., Chiappa V., Raspagliesi F. (2019). Sentinel node mapping in endometrial cancer. Transl. Cancer Res..

